# Quality of Life Score as a Predictor of Death in Dogs with Degenerative Mitral Valve Disease

**DOI:** 10.5935/abc.20170032

**Published:** 2017-04

**Authors:** Célia M. C. Strunz, Mário Marcondes-Santos, Julio Yoshio Takada, Fernanda S. Fragata, Antônio de Pádua Mansur

**Affiliations:** 1 Instituto do Coração (InCor)-HC.FMUSP - Brazil; 2 Hospital Veterinário Sena Madureira, Vila Mariana, São Paulo, SP - Brazil

**Keywords:** Dogs, Quality of Life, Mortality, Heart Valve Diseases, Mitral Valve / abnormalities

## Abstract

**Background:**

The knowledge of the variables predicting mortality is important in clinical practice and for therapeutic monitoring in mitral valve disease.

**Objectives:**

To determine whether a quality of life score evaluated with the Functional Evaluation of Cardiac Health questionnaire would predict mortality in dogs with degenerative mitral valve disease (DMVD).

**Methods:**

Thirty-six client-owned dogs with mitral valve disease underwent clinical, laboratory, and echocardiographic evaluations at baseline and were monitored for 6 months. Cardiovascular death was the primary outcome.

**Results:**

The 36 dogs were classified as survivors or nonsurvivors. Higher values of the following variables were obtained at baseline in the nonsurviving group (12 dogs): amino-terminal pro-B-type natriuretic peptide (NT-proBNP) levels, plasma norepinephrine, heart rate, quality of life score, diastolic left ventricular internal dimension to aortic root ratio, systolic left ventricular internal dimension to aortic root ratio, and left atrium to aortic root ratio. NT-proBNP levels and quality life score were independently associated with death in the multivariable analysis.

**Conclusion:**

The quality life score was an independent variable for cardiac death in dogs with DMVD. This result is encouraging, as this score is easy to apply and does not require any technology, only a veterinarian and an observant owner.

## Introduction

Degenerative mitral valve disease (DMVD) is the most commonly diagnosed disease in routine veterinary cardiology in dogs. Therefore, the knowledge of the variables that can predict mortality in DMVD is important for the clinical practice and for therapeutic monitoring of these patients.^1^

Diagnostic tests, such as electrocardiography, echocardiography, chest radiography, and blood pressure measurement, are routinely used to evaluate these patients and the effectiveness of their treatments.^2^ Other tests have been identified as useful in monitoring the progression of this valvular heart disease. For example, exacerbated activation of the sympathetic nervous system developed during heart failure associated with mitral valve disease can be monitored by measuring the plasma concentration of norepinephrine (NE), which is associated with severe symptoms and a higher risk of death.^3,4^ The importance of the amino-terminal pro-B-type natriuretic peptide (NT-proBNP), an inactive amino-terminal fragment of the prohormone brain natriuretic peptide, has been recognized in recent years. In veterinary medicine, studies in dogs have suggested that NT-proBNP is a marker of the presence and severity of cardiac disease. Cutoff values for the concentrations of this peptide have been established and used to estimate the risk of the onset of congestive heart failure and to predict mortality in dogs with mitral valve disease.^5-7^

In addition to this biochemical marker, the echocardiographic variables left ventricular end-diastolic diameter, left atrial (LA) to aortic root (Ao) ratio (LA/Ao), and E wave transmitral peak velocity are predictors of all-cause mortality in dogs with DMVD.^8^ These diagnostic variables may be used to predict mortality in therapeutic management. Nevertheless, several clinical variables, such as respiratory signs, difficulties with mobility, etc., could together be an important tool to predict death and be very useful in veterinary clinics where technology is unavailable. The aim of this study was to investigate whether a score obtained with the Functional Evaluation of Cardiac Health, a quality of life questionnaire, could be used as a predictor of death in dogs with DMVD.

## Methods

### Animals

The dogs included in this prospective study were referred from a private veterinary ambulatory clinic at the time of their first presentation of signs or symptoms of congestive heart failure. The inclusion criteria for participation in the study were dogs with mitral regurgitation (MR) and LA enlargement (LA/Ao > 1.2) normal laboratory renal (creatinine < 2.1 mg/dL) and liver function results, and no other associated diseases. All dogs underwent a clinical evaluation consisting of physical examination, electrocardiography, blood pressure measurement, thoracic radiography, blood cell count, plasma and serum biochemical analysis, and two-dimensional, M-mode spectral-pulsed Doppler echocardiography. Therapeutic adjustments were only made when the dogs had undergone all diagnostic tests and the quality of life questionnaire had been applied, which occurred after the selection of the animals.

The definitive diagnosis of DMVD was obtained during an echocardiographic examination performed by a veterinary specialist who was blinded to the quality of life questionnaire and laboratory results. The dogs were classified as having grade I to IV DMVD according to the New York Heart Association functional class scoring system modified for veterinary use.^9^ Briefly, functional class I was defined as a heart murmur of mitral origin with no signs of heart enlargement and no limitation to physical activity, class II included slight limitation to physical activity with varying degrees of heart enlargement without clinical signs, class III included marked limitation of physical activity with radiologic signs of congestive heart failure, and class IV comprised severe limitation of physical activity with radiologic signs of congestive heart failure.

Before enrollment in the study, 17 animals were already being treated with diuretics, inotropic agents, and/or angiotensin converting enzyme inhibitors. The drugs administered at the beginning of the study were adjusted according to the severity of the disease and included angiotensin converting enzyme inhibitors, inotropic agents, diuretics, and beta-blockers (when well tolerated). During the 6-month follow-up period, the treatment was adjusted whenever necessary. The owners of the dogs were asked to inform the researcher in case a cardiac-related death occurred outside of the hospital. None of the dogs were euthanized.

An informed written consent was obtained from each dog owner, and the study was approved by the Ethics Committee Heart Institute (InCor), University of São Paulo Medical School (number 072/05).

The variables of the survivors and nonsurvivors at baseline were compared before any therapeutic adjustment was made. The significance of the variables that were clinically relevant in predicting death was analyzed after 6 months of follow-up.

### Assessment of quality of life

A total of 36 client-owned dogs were chosen by convenience for our study. As previously described, the Functional Evaluation of Cardiac Health quality of life questionnaire was developed based on widely accepted clinical signs of cardiac disease in dogs.^10^ The questionnaire consists of 17 questions answered by the dog owner, who grades the severity of symptoms on a scale from 0 to 5, in which 0 = few symptoms and 5 = several symptoms , with higher scores indicating a poorer health-related quality of life. The questions are mainly related to respiratory signs, difficulties with mobility (such as walking and climbing stairs), physical activity, irritability, appetite, sleepiness, and frequency of urination and vomiting. The score was established using information obtained from the owner by a veterinarian during the anamnesis.

### Laboratory measurements

Blood samples were obtained early in the morning for measurement of plasma concentrations of NE, NT-proBNP, and other biochemical variables. An appropriately sized heparinized catheter was inserted into the saphenous vein of each dog. The dog was then placed in lateral recumbency on a table with minimal restraint for 20 minutes.^11^ The first mL of blood collected from the catheter was discarded. The subsequent 3 to 5 mL of blood were collected and immediately transferred to ice-chilled tubes containing a mixture of ethylene glycol tetraacetic acid - glutathione (20 *µ*L of anticoagulant/mL of blood) for NE analysis. Other samples were collected from the same catheter and transferred to an EDTA tube for NT-proBNP measurement and into a plain tube for other biochemical analyses. Within 1 hour of blood collection, the plasma and serum were separated and immediately frozen at -70°C. NE levels were determined by high-performance liquid chromatography with an electrochemical detector,^12^ (Model 515, Waters Corp, Milford, MA, USA) and sodium (Na) levels were analyzed with a selective electrode (Dimension RXL, Dade Behring, Newark, DE, USA). Specific kits for automated equipment were used to measure urea and creatinine levels (Dimension RXL). The concentrations of plasma NT-proBNP were measured in duplicate using a commercial ELISA kit specific for canine NT-proBNP (Vet Sign Canine CardioSCREEN NT- Pro-BNP kit, Guildhay, UK).

### Echocardiographic and electrocardiographic evaluation

The arterial blood pressure was measured indirectly by vascular Doppler (Medmega DV-610, Medmega, São Paulo, Brazil) while the dogs were in lateral recumbency. The cuff width was approximately 40% of the limb circumference. Each systolic and diastolic arterial blood pressure value was calculated as the mean of three to four measurements.

The heart rate (HR) and cardiac rhythm were evaluated using a short-term electrocardiographic recorder (Ecafix model E.C.G.-6, Ecafix , São Paulo, Brazil).^13,14^ The echocardiographic examination was performed using an ultrasound system with a 5-MHz microconvex transducer (Aloka SSD 650 Ultrasound System, Aloka Inc., Tokyo, Japan).

The M-mode echocardiographic variables studied were the diastolic interventricular septal thickness (IVSd), diastolic left ventricular wall thickness (LVWd), diastolic ventricular internal dimension (LVIDd), systolic ventricular internal dimension (LVIDs), fractional shortening (FS), Ao, and LA dimension. The left ventricular dimensions and the LA were indexed to the Ao. FS values were calculated using the equation FS = [(LVIDd - LVIDs) / LVIDd] X 100. The intraobserver variability of the M-mode echocardiographic variables was calculated using 15 measurements of each variable (obtained from three recordings measured five times each) in five dogs (the coefficients of variation ranged from 2.6% to 6.5%).^15^

The severity of the MR was estimated with spectral-pulsed Doppler ultrasonography based on the percentage of the LA occupied by the regurgitant jet (mild < 20%, moderate 20 to 50%, severe > 50%).^16,17^

### Statistical analysis

Data with normal distribution are expressed as mean ± standard deviation (SD), while those with non-normal distribution are shown as median and interquartile range (IQR). The Kolmogorov-Smirnov normality test was used to test for the normality of the data. When the data were normally distributed, the parametric Student's *t* test for independent samples was used, as displayed in Table 1. When the data were not normally distributed, the nonparametric Mann-Whitney *U* test for independent samples (Table 2) and Kruskal-Wallis test (NT-proBNP) were used. In addition, the chi-square test and Fishers' exact test were used when the groups were evaluated in relation to their proportions. The Spearman test was used to measure the statistical association between two variables.

**Table 1:** Baseline characteristics of 36 dogs with degenerative mitral valve disease (DDMV) categorized as survivors or nonsurvivors. Variables with normal distribution, described as mean and standard deviation (SD)

	All	Mortality		
Variables	DMVD dogs (n = 36)	Surviving dogs (n = 24)	Nonsurviving dogs (n = 12)	p
Age (SD) yrs	10.7 (2.0)	10.5 (2.2)	11.0 (1.5)	0.478
Male, n (%)	23 (63.9)	12 (50.0)	11 (91.7)	0.025
FC III-IV, n (%)	15 (41.7)	6 (25.0)	9 (75.0)	0.004
FETCH (SD)	14.9 (10.5)	10.5 (7.9)	23.7 (10.0)	< 0.001
Na (SD) mEq/L	147.0 (4.00)	147.2 (4.19)	146.4 (3.60)	0.550
HR (ECG),(SD),bpm	144.3 (33.8)	137.3 (36.6)	158.3 (22.5)	0.041
SBP (SD) mmHg	135.5 (24.8)	134.8 (26.2)	136.7 (23.0)	0.831
DBP (SD) mmHg	80.1 (16.9)	79.3 (15.3)	81.9 (21.1)	0.689
LVIDd/Ao (SD)	2.23 (0.44)	2.07 (0.39)	2.54 (0.36)	0.0014
LVIDs/Ao (SD)	1.17 (0.28)	1.07 (0.23)	1.36 (0.28)	0.0025
FS (%)	47.7 (6.7)	48.5 (7.0)	45.9 (6.0)	0.277

FC: functional classification; FETCH: Functional Evaluation of Cardiac Health; Na: sodium; HR: heart rate; SBP: systolic blood pressure; DBP: diastolic blood pressure; LVIDd/Ao: diastolic left ventricular internal dimension/aortic root ratio; LVIDs/Ao: systolic left ventricular internal dimension/aortic root ratio; FS: fractional shortening.

**Table 2:** Baseline characteristics of 36 dogs with degenerative mitral valve disease (DDMV) categorized as survivors or nonsurvivors. Variables without normal distribution, described as median and interquartile range (IQR)

	All	Mortality		
Variables	DMVD dogs (n = 36)	Surviving dogs (n = 24)	Nonsurviving dogs (n = 12)	p
Weight (IQR) kg	6.2 (4.5-9.9)	6.2 (4.6-9.9)	6.0 (4.3-10.0)	0.920
NT-proBNP (IQR) pmol/L	1282 (699-2477)	859 (619-1345)	4055 (2070-6452)	< 0.001
NE (IQR) pg/mL	386 (250-574)	293 (214-430)	574 (357-998)	0.017
Creatinine (IQR) mg/dL	0.85 (0.70-1.00)	0.80 (0.70-1.00)	0.90 (0.80-1.10)	0.119
IVSd (IQR) cm	0.60 (0.50-0.70)	0.60 (0.50-0.70)	0.60 (0.50-0.70)	0.890
LA/Ao (IQR)	1.56 (1.38-2.00)	1.44 (1.30-1.65)	2.09 (1.70-2.28)	< 0.001

NT-proBNP: amino-terminal pro-B-type natriuretic peptide; NE: norepinephrine; IVSd: diastolic interventricular septal thickness; LA/Ao: left atrium dimension/aortic root ratio.

We performed a multivariable logistic analysis in a forward stepwise approach considering death at 6 months as the dependent variable. The independent variables were functional classification, LA/Ao, creatinine, quality of life score, ranked NT-proBNP, and dichotomized HR as ≤ 130 bpm or > 130 bpm. NT-proBNP values were ranked in units of 1,000 pmol/L, in order to make it easier to interpret the results.^18^ Only variables with p < 0.1 were included in the multivariable regression model.

Receiver operating characteristic (ROC) analyses were performed to determine the optimal cutoff values for selected variable.^19^ Odds ratios (OR) were calculated as part of the logistic regression analysis. The significance level adopted for the statistical tests was 5%. Statistical analyses were performed using the Statistical Analysis System (SAS) software program for Windows, version 9.2 (SAS Institute Inc., 1989-1996, Cary, NC, USA).

## Results

The following breeds of dogs were enrolled in the study: 23 Poodles, five mixed-breed dogs, one Basset hound, one Beagle, one Cocker Spaniel, one Dachshund, one Lhasa Apso, and three Pinschers. The baseline characteristics of the 36 DMVD dogs are presented in Tables 1 and 2. The dogs were classified as having mild (n = 4), moderate (n = 18), or severe (n = 14) MR.

We investigated the correlation between laboratory, electrocardiographic, echocardiographic, and clinical variables obtained at baseline. A positive correlation was identified between quality of life scores and the following variables: functional classification of the dog (r = 0.729, p < 0.0001), LA/Ao (r = 0.591, p = 0.0001), and plasma NE (r = 0.430, p = 0.009).

NT-proBNP concentrations correlated positively with LA/Ao (r = 0.615, p < 0.001), LVIDd/Ao (r = 0.502, p = 0.0018), and LVIDs/Ao (r = 0.622, p = 0.0001) and negatively with FS (r = -0.386, p = 0.020). The only clinical and biochemical variables that correlated positively with NT-proBNP levels were the quality life score (r = 0.537, p = 0.001) and the plasma NE levels (r = 0.383, p = 0.021).

Dogs with mild (n = 4), moderate (n = 18), and severe (n = 14) MR had NT-proBNP values of 751 pmol/L (IQR 539 - 1017 pmol/L), 1183 pmol/L (IQR 701 - 1850 pmol/L), and 2070 pmol/L (IQR 878 - 5461 pmol/L), respectively (Kruskal-Wallis test, p = 0.0849).

The 36 DMVD dogs were further classified as survivors and nonsurvivors. We compared the clinical, laboratory, and echocardiographic variables of the dogs in both groups to identify factors predictive of death (Tables 1 and 2). The following variables were significantly higher among the animals that did not survive when compared with those that survived: NT-proBNP, NE, HR, quality of life score, LVIDd/Ao, LVIDs/Ao, and LA/Ao. Additionally, most nonsurvival dogs were male (91.7%) and had functional classes III-IV (75.0%).

On multivariable logistic analysis, the variables independently associated with death were NT-proBNP (OR = 2.29, 95% confidence interval [95%CI] 1.24 - 4.2, p = 0.008) and quality of life score (OR = 1.22, 95%CI 1.02 - 1.45, p = 0.027).

The area under the curve, sensitivity, and specificity (obtained from ROC curves) of the univariate models associating NT-proBNP (cutoff = 1850 pmol/L) and the quality of life score (cutoff = 17) with death were 0.91 (95%CI 0.77 - 0.98, standard error [SE] = 0.05, p < 0.0001), 0.83 and 0.88, respectively, and 0.86 (95%CI 0.70 - 0.95, SE = 0.06, p < 0.0001), 0.75 and 0.79, respectively.

**Figure 1:**
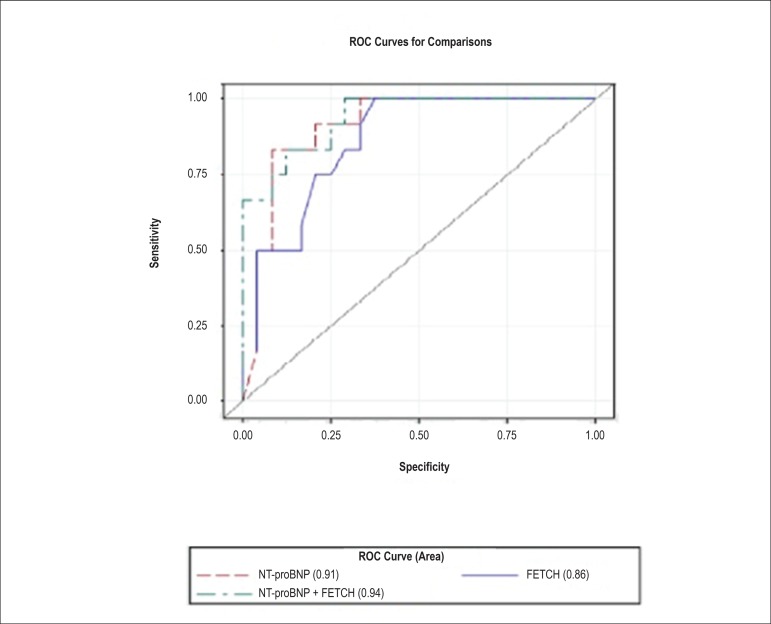
Receiver operating characteristic (ROC) curves for the comparison of the Functional Evaluation of Cardiac Health (FETCH) score and NT-proBNP levels.

Finally, ROC curves were developed for the multivariable model with NT-proBNP and the quality of life score as predictors (Figure 1).

## Discussion

The dogs enrolled in this study had mainly MR, LA enlargement, and preserved renal function. According to our results, the quality of life scores correlated with the functional classification and NE concentrations, while NT-proBNP values correlated with the quality of life score, NE concentrations, and certain echocardiographic findings. On multivariable regression analysis, NT-proBNP concentrations and quality of life score emerged as independent predictors of death after a follow-up period of 6 months. We were also able to calculate the NT-proBNP levels and the quality of life score cutoff values that best predicted mortality.

The association between quality of life scores and NE values with the severity of mitral valve disease has been previously described in the veterinary literature.^3,10^ The positive correlation between these variables suggests that dogs with mitral valve disease that develop heart failure and experience increased sympathetic activity have a decreased quality of life.

The correlation between natriuretic peptide levels and the echocardiographic variables LVIDd/Ao, LVIDs/Ao, LA/Ao, and FS observed in this study have been previously reported by other investigators,^20-22^ confirming that this peptide is a marker of cardiac remodeling and left ventricular dysfunction in dogs with mitral valve disease.

Furthermore, animals with higher concentrations of NT-proBNP or a higher quality of life score had a higher risk of death. The prognostic value of NT-proBNP has been discussed by other investigators. Chetboul et al.^20^ demonstrated the ability of NT-proBNP to predict the transition from asymptomatic mitral insufficiency to a symptomatic disease in dogs. In a prospective study of dogs with symptomatic mitral valve disease over a 6-month follow-up period, Serres et al.^21^ demonstrated that NT-proBNP was a good predictor of survival.

Questionnaires assessing the health-related quality of life of dogs have been validated for a variety of diseases, including cardiac disease, diabetes, neuropathic pain, and skin diseases.^10,23-25^ The questionnaire used in the present study has been already validated in dogs with heart failure.^10^ All studies recommend using the owner-perceived quality of life score for disease management.

In the multivariable regression model, both NT-proBNP concentrations and quality of life score were equally significant and independent predictors of mortality. Still, our most interesting finding was the quality of life score as a predictor of risk of mortality. This result is encouraging, as this questionnaire is easy to apply and does not require any technology, only a veterinarian and an observant owner.

One limitation of our study was the small sample size, which may limit the validity of the results. Another limitation was that the dogs were at different stages of the disease, as shown by their different functional classification. Finally, it is possible that the owner-reported data may have introduced subjectivity into the evaluation.

## Conclusion

The quality of life score was an independent predictor of cardiac death in dogs with DMVD.

## References

[1] Buchanan JW, Fox PR, Sisson D, Moise NS (1999). Prevalence of cardiovascular disorders. Textbook of canine and feline cardiology.

[2] Häggström J, Kvart C, Pedersen HD, Ettinger SJ, Feldman EC (2005). Acquired valvular heart disease. Textbook of veterinary internal medicine.

[3] Ware WA, Lund DD, Subieta AR, Schmid PG (1990). Sympathetic activation in dogs with congestive heart failure
caused by chronic mitral valve disease and dilated
cardiomyopathy. J Am Vet Med Assoc.

[4] Patel MB, Stewart JM, Loud AV, Anversa P, Wang J, Fiegel L (1991). Altered function and structure of the heart in dogs with chronic
elevation in plasma norepinephrine. Circulation.

[5] Moonarmart W, Boswood A, Luis Fuentes V, Brodbelt D, Souttar K, Elliott J (2010). N-terminal pro B-type natriuretic peptide and left ventricular
diameter independently predict mortality in dogs with mitral valve
disease. J Small Anim Pract.

[6] Reynolds CA, Brown DC, Rush JE, Fox PR, Nguyenba TP, Lehmkuhl LB (2012). Prediction of first onset of congestive heart failure in dogs
with degenerative mitral valve disease: the PREDICT cohort
study. J Vet Cardiol.

[7] Wolf J, Gerlach N, Weber K, Klima A, Wess G (2012). Lowered N-terminal pro-B-type natriuretic peptide levels in
response to treatment predict survival in dogs with symptomatic mitral valve
disease. J Vet Cardiol.

[8] Borgarelli M, Savarino P, Crosara S, Santilli RA, Chiavegato D, Poggi M (2008). Survival characteristics and prognostic variables of dogs with
mitral regurgitation attributable to myxomatous valve
disease. J Vet Intern Med.

[9] Sisson D, Kittleson MD, Fox PR, Sisson D, Moïse NS (1999). Management of heart failure: principles of treatment,
therapeutics strategies and pharmacology. Textbook of canine and feline cardiology - principles and clinical
practice.

[10] Freeman LM, Rush JE, Farabaugh AE, Must A (2005). Development and evaluation of a questionnaire for assessing
health-related quality of life in dogs with cardiac disease. J Am Vet Med Assoc.

[11] Bühler HU, da Prada M, Haefely W, Picotti GB (1978). Plasma adrenaline, noradrenaline and dopamine in man and
different animal species. J Physiol.

[12] Bouloux P, Perrett D, Besser GM (1985). Methodological considerations in the determination of plasma
catecholamines by high-performance liquid chromatography with
electrochemical detection. Ann Clin Biochem.

[13] Edwards NJ (1987). Bolton's handbook of canine and feline electrocardiography.

[14] Tilley LP (1992). Essentials of canine and feline electrocardiography.

[15] Chetboul V, Tidholm A, Nicolle A, Sampedrano CC, Gouni V, Pouchelon JL (2005). Effects of animal position and number of repeated measurements on
selected two-dimensional and M-mode echocardiographic variables in healthy
dogs. J Am Vet Assoc.

[16] Boon JA (1998). Manual of veterinary echocardiography.

[17] Brown DJ, Rush JE, MacGregor J, Ross JN Jr, Brewer B, Rand WM (2003). M-mode echocardiographic ratio indices in normal dogs, cats, and
horses: a novel quantitative method. J Vet Intern Med.

[18] Masson L, Latini R, Anand IS, Vago T, Angelici L, Barlera S, Val-HeFT investigators (2006). Direct comparison of B-type natriuretic peptide (BNP) and
amino-terminal proBNP in a large population of patients with chronic and
symptomatic heart failure: the Valsartan Heart Failure (Val-HeFT)
data. Clin Chem.

[19] Youden WJ (1950). Index for rating diagnostic tests. Cancer.

[20] Chetboul V, Serres F, Tissier R, Lefebvre HP, Sampedrano CC, Gouni V (2009). Association of plasma N-terminal pro-B-type natriuretic peptide
concentration with mitral regurgitation severity and outcome in dogs with
asymptomatic degenerative mitral valve disease. J Vet Internal Med.

[21] Serres F, Pouchelon JL, Poujol L, Serres F, Pouchelon JL, Poujol L (2009). Plasma N-terminal pro-B-type natriuretic peptide concentration
helps to predict survival in dogs with symptomatic degenerative mitral valve
disease regardless of and in combination with the initial clinical status at
admission. J Vet Cardiol.

[22] Takemura N, Toda N, Miyagawa Y, Asano K, Tejima K, Kanno N (2009). Evaluation of plasma N-terminal Pro-Brain natriuretic peptide
(NT-proBNP) concentrations in dogs with mitral valve
insufficiency. J Vet Med Sci.

[23] Noli C, Colombo S, Cornegliani L, Ghibaudo G, Persico P, Vercelli A (2011). Quality of life of dogs with skin disease and of their owners.
Part 2: administration of a questionnaire in various skin diseases and
correlation to efficacy of therapy. Vet Dermatol.

[24] Niessen SJ, Powney S, Guitian J, Niessen AP, Pion PD, Shaw JA (2012). Evaluation of a quality-of-life tool for dogs with diabetes
mellitus. J Vet Intern Med.

[25] Rutherford L, Wessmann A, Rusbridge C, McGonnell IM, Abeyesinghe S, Burn C (2012). Questionnaire-based behaviour analysis of Cavalier King Charles
spaniels with neuropathic pain due to Chiari-like malformation and
syringomyelia. Vet J.

